# Dihydromyrcenol Modulates Involucrin Expression through the Akt Signaling Pathway

**DOI:** 10.3390/ijms25042246

**Published:** 2024-02-13

**Authors:** Suhjin Yang, Wesuk Kang, Dabin Choi, Jiyun Roh, Taesun Park

**Affiliations:** Department of Food and Nutrition, BK21 FOUR, Yonsei University, 50 Yonsei-ro, Seodaemun-gu, Seoul 120-749, Republic of Korea; ysj765@naver.com (S.Y.); wesuk42@naver.com (W.K.); vin1411@naver.com (D.C.); y20311@naver.com (J.R.)

**Keywords:** dihydromyrcenol, involucrin, Akt, keratinocyte, skin barrier

## Abstract

The epidermis serves as a protective barrier against external threats and is primarily composed of keratinocytes, which ultimately form corneocytes. Involucrin, a protein integral to the cornified envelope, plays a pivotal role in preserving the functional integrity of the skin barrier. Previous studies have shown that Akt plays an important role in keratinocyte differentiation and skin barrier development. This study investigated whether dihydromyrcenol (DHM), a plant-derived terpene, could increase involucrin production in keratinocytes and sought to elucidate the possible underlying mechanisms. To accomplish this objective, we assessed the alterations in involucrin by DHM through quantitative PCR and Western blot on the HaCaT cell line. The changes in the promoter levels were investigated using luciferase assays. Furthermore, upstream mechanisms were explored through the use of siRNA and inhibitors. To strengthen our findings, the results were subsequently validated in primary cells and 3D skin equivalents. DHM significantly increased involucrin mRNA and protein levels in a concentration-dependent manner. In addition, the Fyn-Akt signaling pathway was found to be required for DHM-induced involucrin expression, as inhibition of Fyn or Akt blocked the increase in involucrin mRNA induced by DHM. The transcription factor Sp1, which is recognized as one of the transcription factors for involucrin, was observed to be activated in response to DHM treatment. Moreover, DHM increased epidermal thickness in a 3D human skin model. These findings suggest that the modulation of involucrin expression with DHM could improve skin barrier function and highlight the importance of manipulating the Akt pathway to achieve this improvement.

## 1. Introduction

The epidermis, or outer layer of the skin, serves as a barrier to protect the body from external pathogens, allergens, and water loss; thus, it is also called the skin barrier. This barrier is composed of keratinocytes, which eventually differentiate into corneocytes, forming a cornified lipid envelope in the outermost layer of the skin. During this process, cornified envelope proteins, such as involucrin, filaggrin, and loricrin, are synthesized and contribute to the structure of the skin barrier through their association with intercellular lipids, including ceramides and fatty acids [1,2,3].

Conditions such as skin aging, delayed wound healing, and severe skin diseases such as psoriasis and dermatitis often result from barrier disruption. In the past, such disruptions were attributed to internal abnormalities, including the breakdown of the immune system. However, the discovery of barrier proteins has led to the understanding that these proteins can be directly regulated, giving rise to strategies targeting skin barrier proteins at the cellular level [4,5,6]. This shift in understanding has prompted several recent studies to explore natural substances capable of modulating barrier proteins and, subsequently, barrier function [7,8,9]. Despite these efforts, it appears that developments in this field are still at an early stage, and there are currently few natural compounds that are widely accepted for increasing skin barrier proteins.

Akt, a serine/threonine kinase, has been extensively studied because of its role in numerous intracellular signaling pathways implicated in cell growth, survival, and differentiation [10,11]. Based on its biological function, this kinase is expected to play a pivotal role in skin barrier development. In the early stages of research, suppression of Akt phosphorylation through a specific inhibitor was shown to impair cornified protein production, and Akt-knockout animals exhibited severe destruction of the skin barrier, providing solid evidence of a link between Akt and barrier development [12,13,14]. Recent studies have focused on identifying the upstream regulators of Akt as potential targets for stimulating barrier protein synthesis. For example, both leucine-rich repeat LGI family member 3 and epiprofin have been identified as upstream effectors of the Akt pathway, instigating the production of cornified envelope proteins [15,16].

Among the aforementioned cornified envelope proteins, involucrin is crucial for maintaining the skin barrier, as it provides significant structural integrity through its glutamine-rich tandem repeats, which cross-link with other structural proteins and lipids. Notably, involucrin is either scarce or absent in poorly differentiated keratinocytes. Therefore, involucrin is considered to be a pivotal factor in preserving the functional integrity of the skin barrier and is a marker of cornified envelope production [5,17,18,19]. In our pilot experiments, we used the luciferase assay with the involucrin promoter to quickly test natural compounds from our phytochemical library consisting of approximately 500 compounds. To ensure optimal efficiency, these screenings were carried out at a constant concentration of 200 µM with a single iteration. Importantly, dihydromyricetin (DHM) exhibited a remarkable increase in the activity of the involucrin promoter, suggesting its potential to enhance barrier molecules. DHM is a terpene commonly found in plants such as *Citrus japonica* and *Ribes nigrum*. It has been extensively used commercially as a fragrance, including in bath products, owing to its pleasant orange-like scent. The goal of this study was to determine whether DHM increases involucrin levels in keratinocytes strengthens the skin barrier, and identify the potential mechanisms involved.

## 2. Results

### 2.1. DHM Did Not Affect the Viability of HaCaT Cells at Concentrations up to 200 μM

**Figure 1:**
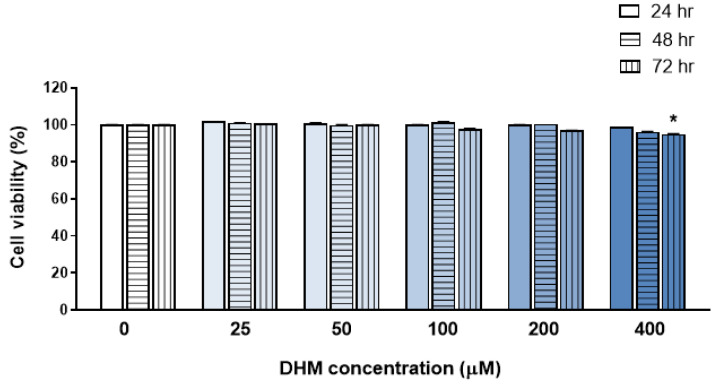
DHM did not affect the viability of HaCaT cells at concentrations up to 200 μM. HaCaT cells were treated with various concentrations of dihydromyrcenol (DHM; 25, 50, 100, 200, and 400 μM) for 24, 48, and 72 h. Cell viability was assessed using a cell count kit-8 (CCK-8) assay. Histograms indicate the percentage of viable cells relative to the vehicle (dimethyl sulfoxide; DMSO)-treated control. Data are presented as mean ± standard error of the mean (SEM) from three independent experiments. * *p* < 0.05.

To investigate the cytotoxicity of DHM, HaCaT cells were treated with various concentrations of DHM (25, 50, 100, 200, and 400 µM). Cell viability was assessed at 24, 48, and 72 h post-treatment using the cell count kit-8 (CCK-8) assay, with untreated cells serving as controls. As shown in Figure 1, no cytotoxic effects were observed in HaCaT cells up to a DHM concentration of 200 µM at any time point. However, a significant decrease, of approximately 5%, in cell viability was observed after 72 h of treatment with 400 µM of DHM. Consequently, for subsequent experiments, DHM was used at concentrations not exceeding 200 µM.

### 2.2. DHM Increased Involucrin Expression in HaCaT Cells

**Figure 2:**
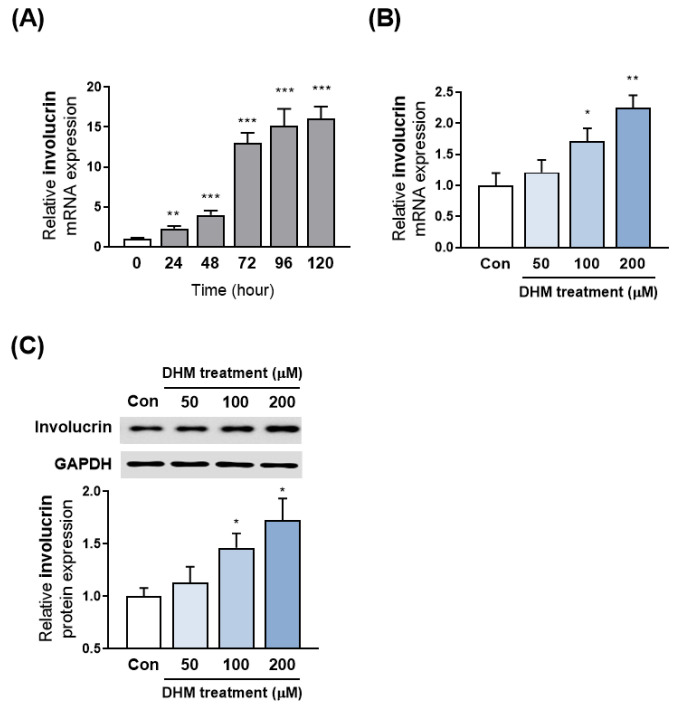
DHM increased involucrin expression in HaCaT cells. (**A**) The levels of involucrin mRNA during keratinocyte differentiation, induced by replacing the medium with a high calcium concentration (1.8 mM), were determined at various time points (24, 48, 72, 96, and 120 h) using quantitative reverse transcription PCR (RT-qPCR). (**B**) HaCaT cells were treated with different concentrations of DHM (50, 100, and 200 μM) for 48 h during the differentiation process, and involucrin expression was assessed using RT-qPCR. (**C**) The effect of DHM treatment on involucrin protein expression was evaluated by Western blot analysis after exposure to DHM at 50, 100, and 200 μM for 48 h, with glyceraldehyde 3-phosphate dehydrogenase (GAPDH) serving as an internal control. Data are presented as the mean ± SEM, derived from three independent experiments. * *p* < 0.05; ** *p* < 0.01; *** *p* < 0.001.

Given the critical role of involucrin in directing keratinocyte differentiation and its specific expression in the differentiating epidermis, we first investigated involucrin expression under conditions that enhance keratinocyte differentiation. For this purpose, we incubated HaCaT keratinocytes in a differentiation medium at various time points: 24, 48, 72, 96, and 120 h. We observed that involucrin mRNA levels progressively increased over time, with a notable surge beginning 48 h post-differentiation induction and plateauing around 96 h (Figure 2A). To evaluate the stimulatory effect of DHM on involucrin expression, keratinocytes were exposed to DHM in a differentiation medium for a period of up to 48 h before involucrin levels had fully increased due to the differentiation medium alone. A timeline depicting the experimental designs of the current study is shown in Figure S1. Upon administering varying concentrations of DHM to differentiating HaCaT cells for 48 h, we observed a significant, concentration-dependent, increase in involucrin mRNA levels compared to those in keratinocytes that were incubated solely in a differentiation medium (Figure 2B). Additionally, we assessed the involucrin protein expression level using Western blotting after treating the cells with DHM at concentrations of 50, 100, and 200 μM for 48 h. Our results confirmed that DHM treatment significantly increased involucrin protein levels (Figure 2C). Furthermore, we examined the effects of DHM on two other well-known cornified proteins, filaggrin and loricrin. Our analysis revealed that a concentration of 200 μM of DHM enhanced filaggrin expression, but did not significantly alter the mRNA expression of loricrin in HaCaT keratinocytes (Figure S2).

### 2.3. Akt Is Required for the DHM-Mediated Induction of Involucrin in HaCaT Cells

**Figure 3:**
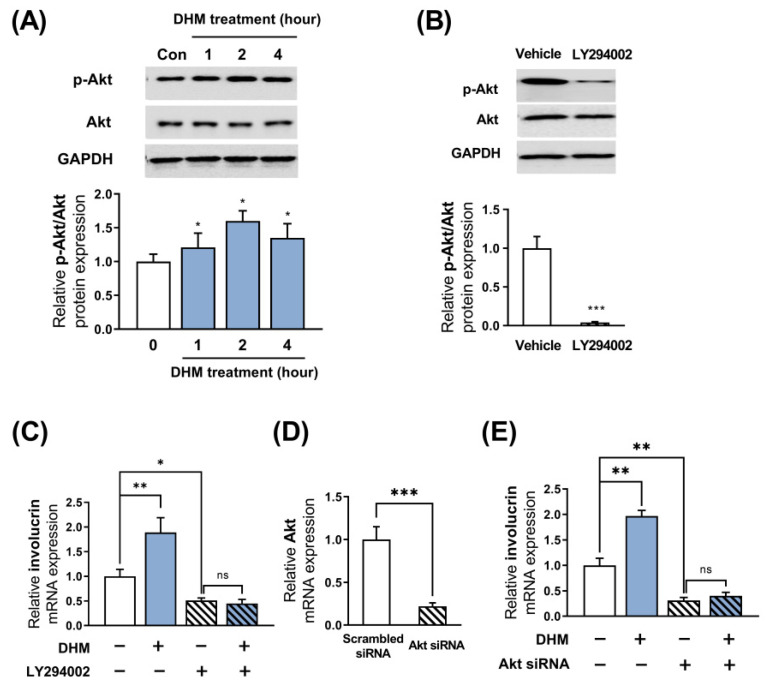
Akt is required for the DHM-mediated induction of involucrin in HaCaT cells. (**A**) Western blot was performed at various time intervals to examine Akt phosphorylation (p-Akt) induced by DHM (200 μM) in HaCaT cells. (**B**) Western blot analysis of p-Akt levels in HaCaT cells after treatment with vehicle (DMSO) or LY294002 (50 μM) for 1 h. (**C**) Cells were pretreated with LY294002 (50 μM) for 1 h and subsequently incubated with DHM (200 μM) for 48 h. Following this, RT-qPCR was performed to measure involucrin mRNA expression. (**D**) Cells were transfected with Akt siRNA. 48 h after transfection, mRNA levels of Akt were determined by qRT-PCR. (**E**) Akt knockdown cells were exposed to DHM (200 μM) for a duration of 48 h, after which the mRNA levels of involucrin were measured. Data are presented as mean ± SEM, derived from three independent experiments. * *p* < 0.05; ** *p* < 0.01; *** *p* < 0.001; ns, not significant.

A growing body of experimental evidence suggests that Akt is crucially implicated in involucrin expression in HaCaT keratinocytes. To investigate whether DHM could indeed phosphorylate and activate Akt in differentiating keratinocytes, we performed Western blot analysis to examine the phosphorylation levels of Akt. We have previously observed modifications in the mRNA of involucrin at the 48 h time point following DHM treatment. Considering that the phosphorylation of phosphoproteins, such as Akt, is a crucial signaling mechanism that influences gene alterations, typically taking place within a few hours, our focus was directed towards assessing Akt phosphorylation within the 1 to 4 h timeframe subsequent to DHM treatment. We observed marked phosphorylation in Akt, reaching a peak at 2 h after DHM treatment (Figure 3A). Accordingly, we selected a 2 h treatment time with DHM for further mechanistic analysis, aiming to elucidate the role of the Akt pathway in the effects of DHM. We then sought to determine whether the activation of Akt by DHM contributes to the induction of involucrin transcription by inhibiting the Akt signaling pathway using its specific inhibitor, LY294002. Initially, we verified that Akt phosphorylation was significantly suppressed following the treatment of HaCaT cells with LY294002 (50 μM) (Figure 3B). The DHM-mediated upregulation of involucrin was evaluated with or without LY294002 (50 μM). We found that LY294002 not only significantly decreased involucrin expression, but also completely negated the DHM-induced upregulation of involucrin in HaCaT cells (Figure 3C). To establish with certainty the involvement of Akt in the expression of involucrin induced by DHM, the effects of Akt blockade were validated using gene-silencing techniques, specifically siRNA. HaCaT cells were transiently transfected with Akt siRNA or scrambled siRNA as a negative control. After a 48 h period, we evaluated the expression levels of Akt mRNA and found that the transfection of Akt siRNA effectively suppressed mRNA levels of Akt in HaCaT cells (Figure 3D). Subsequently, Akt knockdown HaCaT cells were subjected to an additional 48 h of DHM treatment. The results indicated that the knockdown of Akt nullified the effect of DHM in HaCaT cells; similar to the trend observed with the specific inhibitor LY294002 (Figure 3E). This suggests that targeting Akt using either an inhibitor or siRNA yields significant results. Therefore, in the subsequent experiments, the Akt inhibitor approach was representatively employed to investigate the role of Akt. While LY294002 itself reduced filaggrin expression in the presence of DHM, it failed to inhibit the stimulatory effect of DHM on filaggrin expression (Figure S3). These findings suggest that the Akt signaling pathway is closely associated with the upregulated expression of involucrin in response to DHM treatment.

### 2.4. Fyn Serves as a Potential Upstream Regulator of DHM-Induced Akt Phosphorylation and Involucrin Expression in HaCaT Cells

**Figure 4:**
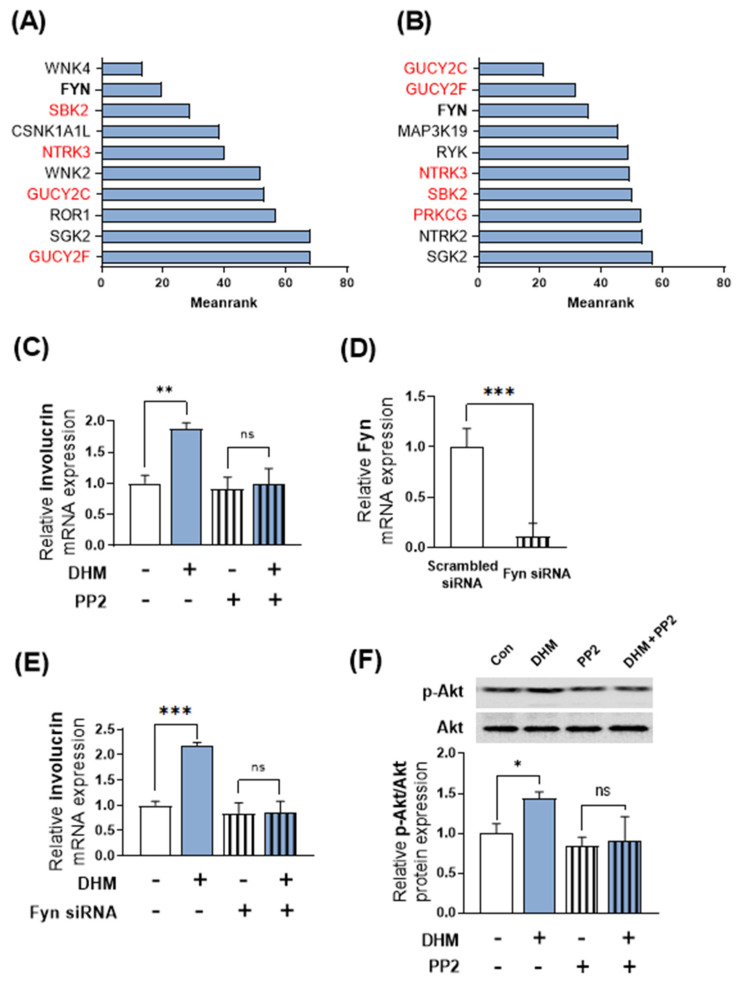
Fyn serves as a potential upstream regulator of DHM-induced Akt phosphorylation and involucrin expression in HaCaT cells. HaCaT cells were treated with either vehicle or DHM (200 μM) for 24 h and subsequently subjected to RNA sequencing using next-generation sequencing technology. Genes exhibiting a minimum of (**A**) 1.5- or (**B**) 2-fold increase in mRNA expression following DHM treatment, as compared to the vehicle control, were identified. A comprehensive bioinformatics analysis utilizing the Kinase Enrichment Analysis 3 tool was conducted on this selected set of genes. The MeanRank score was used to rank the kinases, and the top ten kinases are listed in the figures. The lower value indicates a higher degree of significant enrichment for upstream kinases. Any kinase scarcely expressed in keratinocytes (read counts in RNA-Seq data equal to zero) is marked in red. (**C**) HaCaT cells were exposed to a Fyn kinase inhibitor (PP2, 10 µM) for a period of 1 h. Subsequently, the impact of this inhibition on involucrin expression in response to DHM treatment was evaluated over a duration of 48 h. (**D**) The cells were transfected with Fyn siRNA. After 48 h of transfection, the levels of Fyn mRNA were determined using qRT-PCR. (**E**) Fyn knockdown cells were subjected to DHM treatment (200 μM) for 48 h, following which the levels of involucrin mRNA were measured. (**F**) Before incubation with DHM (200 μM) for 2 h, HaCaT cells were pretreated with a Fyn inhibitor (PP2) for 1 h. The levels of phospho-Akt and total Akt were subsequently assessed through Western blot analysis. Data are presented as mean ± SEM from a representative experiment performed in triplicate. * *p* < 0.05; ** *p* < 0.01; *** *p* < 0.001; ns, not significant.

Given that Akt is modulated by several upstream factors, we hypothesized that DHM initially targets specific kinases that serve as upstream partners of Akt, resulting in subsequent Akt activation. Therefore, we aimed to identify the upstream kinases responsible for the DHM-induced Akt phosphorylation. To elucidate the potential upstream regulatory mechanisms underlying the observed changes in Akt phosphorylation by DHM, we employed a recently developed bioinformatics tool, Kinase Enrichment Analysis 3 (KEA3). KEA3 is a bioinformatics tool that enables researchers to infer the overrepresentation of upstream kinases responsible for the upregulation of genes or proteins. First, we conducted next-generation sequencing (NGS) analysis and selected gene profiles that were upregulated by 1.5- and 2-fold following DHM treatment in HaCaT cells. We input these profiles into the KEA tool and extracted the top-ranked most enriched kinases associated with DHM treatment. We then excluded proteins that were rarely expressed in HaCaT cells based on the NGS data. Consequently, we identified Fyn as a potential upstream regulator that predominantly mediates the effects of DHM, as it repeatedly appeared at the top of both lists (Figure 4A,B). To confirm this bioinformatic analysis, we evaluated the effect of DHM under Fyn-inhibited conditions and observed that the Fyn inhibitor, PP2 (10 μM), nullified the DHM-induced upregulation of involucrin expression (Figure 4C). To validate the involvement of Fyn in the expression of involucrin induced by DHM, similar to the case of Akt, we utilized the siRNA technique to inhibit Fyn and confirm its effects. Initially, HaCaT cells were transfected with Fyn siRNA or scrambled siRNA as a negative control. Subsequently, we evaluated the expression levels of Fyn mRNA in HaCaT cells after 48 h and verified the marked suppression of Fyn mRNA levels by Fyn siRNA (Figure 4D). Following this, Fyn knockdown HaCaT cells were subjected to treatment with DHM for an additional 48 h. The results demonstrated that the knockdown of Fyn abolished the impact of DHM in HaCaT cells; similar to the trend observed with the Fyn inhibitor PP2 (Figure 4E). Consequently, this indicates that targeting Fyn, whether through an inhibitor or siRNA, yields significant results. Therefore, in the subsequent experiments, we representatively utilized the Fyn inhibitor to investigate the role of Fyn. Next, we treated HaCaT cells with DHM under conditions of Fyn inhibition using the PP2 and quantified changes in Akt phosphorylation levels via Western blot analysis. The measurement of Akt phosphorylation was performed at a representative time point of 2 h, which had previously been identified as displaying the most significant alterations induced by DHM. Intriguingly, the effect of DHM on Akt protein phosphorylation was effectively negated under Fyn-inhibited conditions, suggesting that Fyn potentially operates upstream of Akt signaling (Figure 4F). Thus, our findings support the hypothesis that Fyn acts upstream of the Akt pathway and mediates the induction of involucrin transcription by DHM in HaCaT keratinocytes.

### 2.5. Sp1 Is Implicated as the Downstream Effector of Akt in Response to DHM

**Figure 5:**
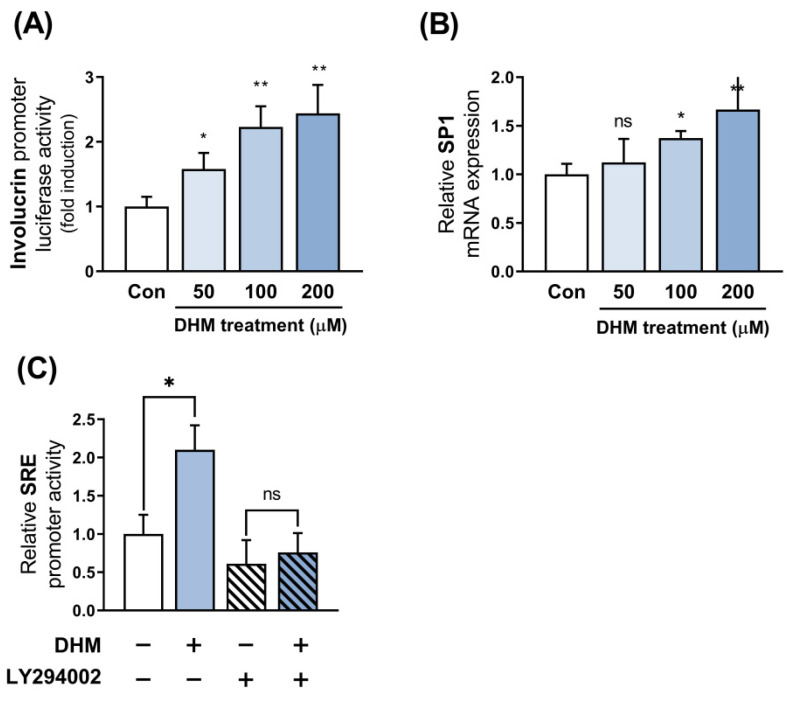
Sp1 is implicated as the downstream effector of Akt in response to DHM. (**A**) Involucrin promoter activity was assessed using a dual luciferase assay after exposure to varying concentrations (50, 100, and 200 μM) of DHM for 48 h in HaCaT cells. (**B**) Sp1 mRNA levels were measured by RT-qPCR after treatment of HaCaT cells with different concentrations of DHM for 12 h. (**C**) The Sp1 response element (SRE) promoter assay was conducted to evaluate the impact of DHM treatment (200 μM) for 48 h in HaCaT cells. If required, HaCaT cells were pretreated with an Akt inhibitor (LY294002; 50 μM) for 1 h before incubation with DHM (200 μM) for 48 h, and the SRE promoter activity was assessed using a dual luciferase assay. Data represent mean ± SEM from three independent experiments. * *p* < 0.05; ** *p* < 0.01; ns, not significant.

In contrast to our previous experiment, which aimed to explore the potential upstream effectors of Akt, we focused on determining the downstream effectors of Akt involved in the DHM-mediated upregulation of involucrin. Initially, we investigated whether DHM regulates involucrin expression at the promoter level by cloning the promoter region of the human involucrin gene. We then evaluated the activity of this promoter in response to DHM treatment in HaCaT cells using a dual luciferase assay. Since the quantity of involucrin transcript was measured at 48 h, the promoter activity directly related to its production was also evaluated at the same time point. Consistent with our mRNA and protein findings, we found that DHM enhanced the activity of the involucrin promoter in a dose-dependent manner (Figure 5A). Subsequently, we sought to identify the upstream transcription factors responsible for regulating involucrin promoter activity in response to DHM treatment. Next, we undertook further analyses to ascertain whether this transcription factor, Sp1, followed the same trend as involucrin promoter activity. Given that the promoter activity of involucrin exhibited changes at 48 h, we anticipated that the increase in the transcription factor responsible for its regulation would occur earlier, along with an earlier occurrence of its mRNA. Moreover, considering that Akt phosphorylation was observed for up to 4 h, we selected 12 h as an intermediary time point to measure the mRNA of Sp1, the putative transcription factor of involucrin. As hypothesized, Sp1 mRNA levels paralleled the increase in involucrin levels following DHM treatment (Figure 5B). To confirm this hypothesis, we performed an Sp1 response element (SRE) promoter assay. DHM treatment amplified SRE promoter activity, suggesting its role in boosting the transcription of genes regulated by Sp1. We then verified that DHM-induced SRE activation was inhibited by LY294002, indicating that Sp1 activation resulted from Akt-mediated action (Figure 5C). These findings, alongside the existing literature, suggest the potential involvement of Sp1 in the molecular mechanism of DHM.

### 2.6. DHM Enhances Involucrin Expression in Primary Keratinocytes via the Fyn-Akt Pathway

**Figure 6:**
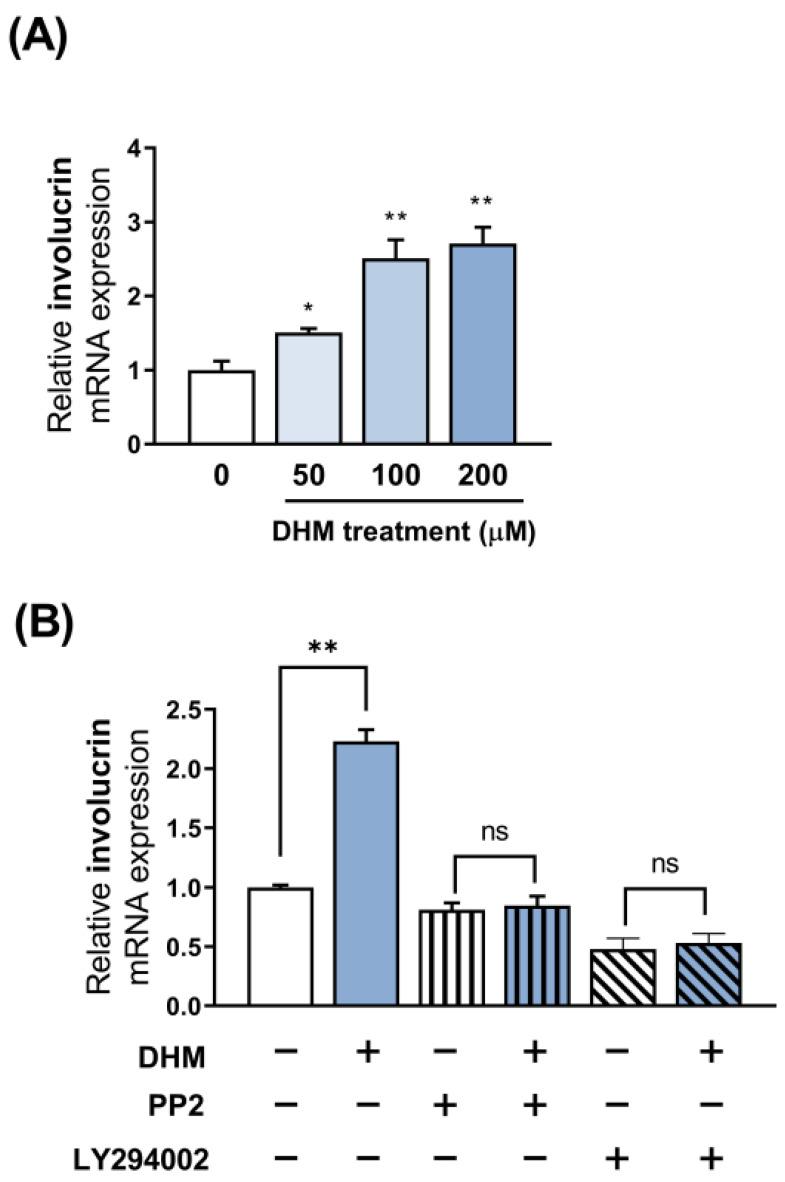
DHM enhances involucrin expression in primary keratinocytes via the Fyn-Akt pathway. (**A**) Primary NHEK cells were treated with increasing concentrations of DHM (50–200 µM), and involucrin mRNA levels were assessed using RT-qPCR. (**B**) NHEK cells were pretreated with either a Fyn inhibitor (PP2, 10 µM) or an Akt inhibitor (LY294002, 50 µM) for 1 h, followed by treatment with DHM (200 µM) for 48 h. The normalized value of involucrin mRNA relative to that of GAPDH was evaluated. Each data point represents the mean ± SEM of three independent experiments. * *p* < 0.05; ** *p* < 0.01; ns, not significant.

To date, our observations have focused on DHM-induced involucrin expression in the HaCaT keratinocyte cell line. These findings led us to investigate whether DHM could produce a similar effect in primary keratinocytes and not merely in a specific established cell type. To verify this hypothesis, we first applied DHM to normal human epidermal keratinocytes (NHEK) to ascertain the potential beneficial effects of this compound on primary cells. The NHEKs were exposed to the exact same DHM concentrations (ranging from 50 to 200 μM) as the HaCaT cells. We then analyzed involucrin mRNA expression levels using quantitative reverse transcription PCR (RT-qPCR). Consistent with the findings in HaCaT cells, involucrin expression levels in NHEKs increased in a DHM concentration-dependent manner. Specifically, at 200 μM DHM treatment, the level of involucrin mRNA increased to a maximum of approximately two times that of the control, similar to that of involucrin mRNA observed in HaCaT cells, suggesting that the promoting effects of DHM on involucrin expression are likely to be commonly observed in human keratinocytes (Figure 6A). We then sought to determine whether DHM also influences NHEKs via the Fyn-Akt pathway, mirroring the previously observed impact on HaCaT cells. To this end, we inhibited either the Fyn or Akt pathway using their respective inhibitors, PP2 or LY294002. We discovered that the enhancing effects of DHM on involucrin expression disappeared after NHEKs were treated with the Fyn inhibitor. Consistent with this, we demonstrated that blocking Akt signaling with LY294002 completely abolished the promotion of DHM-induced involucrin transcription (Figure 6B). Collectively, these data indicate that DHM may induce involucrin expression in general keratinocytes, and this effect appears to be mediated through the Fyn-Akt pathway.

### 2.7. DHM Promoted Barrier Formation through the Fyn-Akt Pathway in a 3D Human Skin Model

**Figure 7:**
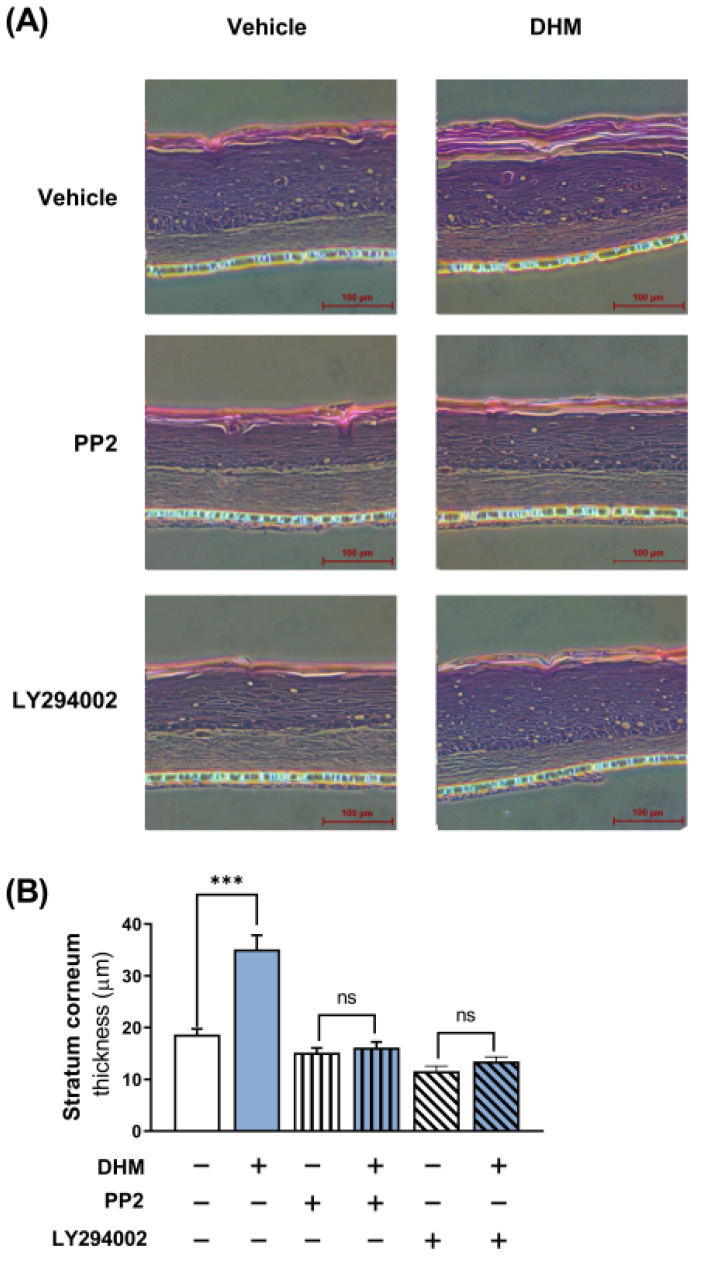
DHM promoted barrier formation through the Fyn-Akt pathway in a 3D human skin model. The human skin equivalent (HSE) model was treated with DHM, PP2, or LY294002 and then incubated for 4 days. HSE was subsequently fixed, embedded in paraffin, sectioned, and stained with Hematoxylin and Eosin. (**A**) Representative images captured under a microscope were presented. (**B**) Epidermal thickness was measured. The data represent the mean ± SEM of three independent experiments. *** *p* < 0.001; ns, not significant.

The established role of involucrin in skin barrier development prompted us to investigate the potential contribution of DHM to this process at the organ-tissue level. However, studies on barrier formation are largely restricted to in vitro conditions, as keratinocytes typically grow as monolayers in culture. To address this limitation, we employed a 3D skin model, specifically, a model of the reconstructed human epidermis (EpiDerm), to evaluate the effects of DHM and discern its underlying mechanism of action. We measured the epidermal thickness in the presence and absence of Fyn- or Akt-specific inhibitors. To further ascertain the thickness of the epidermis, which is used as a marker of barrier development, we applied hematoxylin and eosin staining post-DHM treatment, both with and without the aforementioned inhibitors. The results of our analysis illustrated that the thickness of the epidermis in the DHM-treated skin model was significantly greater than that in the vehicle control. Furthermore, we confirmed that DHM failed to exert its thickening effects in the presence of an Akt or Fyn inhibitor, corroborating our previous in vitro observations (Figure 7). In summary, the beneficial effect of DHM is demonstrably apparent in 3D human skin, seemingly causing a significant acceleration in normal epidermal barrier formation.

## 3. Discussion

Numerous studies have demonstrated that the Akt pathway regulates involucrin expression; however, the direction of this regulation remains inconsistent. Some studies have reported an increase in involucrin levels when the Akt pathway is activated, whereas others have reported a decrease [15,20,21,22,23]. Although the factors that regulate this direction remain largely unknown, it is important to note that researchers have scrutinized the Akt pathway for its delicate balance of a variety of interrelated cellular processes, including cell proliferation, survival, and differentiation, and the extent of Akt activation may determine the outcome of a specific event. For instance, appropriate levels of Akt activation in keratinocytes have been suggested to promote growth arrest and differentiation, which facilitates barrier formation. However, excessive activation of Akt signaling has been reported in relation to excessive proliferation and defective keratinocyte differentiation, which leads to a decrease in the production of barrier proteins [24,25,26]. In our study, we observed that treatment with DHM caused a modest increase in Akt phosphorylation, which seemed to be sufficient, but not excessive, to elevate involucrin expression. Further research is crucial to fully understand what determines the regulatory direction of the Akt pathway in involucrin expression, and how these effects might differ in various cellular contexts.

In this study, two concerns arise regarding the application of DHM. Firstly, our findings demonstrate that, while DHM exhibits significant biological effects at concentrations of 100 μM or higher, attaining these levels systemically in vivo may not be easily achievable. Secondly, DHM exhibits its biological effects involving Akt-dependent Sp1 activation. This mechanism, while effective, may also intersect with pathways that have pro-oncogenic potential [27,28]. To address these issues, we would like to emphasize that DHM should primarily be considered for topical application. This approach offers unique advantages, as it enables the attainment of higher local concentrations at the site of application, potentially reaching the effective levels observed in our experiments. Furthermore, such localized application mitigates the risks associated with systemic exposure, particularly concerning the pro-oncogenic potential of DHM. It is widely recognized that compounds with effective skin permeation typically possess a low molecular weight (below 500 Da) and are lipophilic [29]. DHM, with its lipophilic properties and a molecular weight of 156.3 Da, fulfills these criteria and is thus likely to be readily absorbed through the skin, efficiently reaching the epidermis. Additionally, it is noteworthy that DHM has already been employed in the cosmetic industry as a fragrance ingredient, where its safety profile has been established without any reported instances of skin carcinogenesis, irritation, or sensitization associated with its use in these contexts. These data provide further support for the potential of DHM in topical therapeutic applications.

Akt is a protein that can be regulated by various upstream factors. To understand the mechanisms underlying this regulation, we used bioinformatics analysis of RNA sequencing data to explore the potential upstream regulators of Akt. Our results suggest that FYN, an Src family tyrosine kinase, is the most likely candidate for the regulation of Akt by DHM. In line with the current bioinformatic findings, previous studies have frequently indicated that FYN is a potential factor that increases Akt activity in mammalian cells, including keratinocytes. FYN has been reported to activate the Akt pathway either through the phosphorylation of tyrosine residues on upstream receptors or by direct phosphorylation of Akt [30,31,32,33]. In this study, we found that the inhibition of FYN negated the effect of DHM on Akt phosphorylation, thus supporting the idea that FYN may play a pivotal role in the Akt regulatory system within keratinocytes.

FYN, a member of the Src family of tyrosine kinases, is a non-receptor protein tyrosine kinase that encompasses an array of domains pivotal to its functionality. Notably, the Src Homology (SH) domains, specifically the SH2 and SH3 domains, operate as the principal functional units within FYN. These domains facilitate interactions with other molecules, thereby mediating a myriad of cellular processes [34,35,36]. Our investigations indicate that DHM, a type of phytochemical, potentiates the effects of involucrin via FYN. However, the mechanisms through which DHM modulates these domains remain unclear. The factors that activate FYN are not well characterized and remain a subject of ongoing research. Nonetheless, several reports have suggested that phytochemicals, such as myricetin, caffeic acid, and delphinidin, may activate FYN. These phytochemicals directly bind to FYN and cause changes in its kinase activity [37,38,39,40]. In contrast, phytochemicals such as sulforaphane appear to stimulate FYN through the modulation of other upstream signaling pathways, including glycogen synthase kinase-3β [41]. Further investigation is needed to fully understand the mechanism by which DHM targets FYN and to identify the specific part of FYN that it affects.

Previous research studies have indicated that the promoter region of involucrin can be activated by Sp1 [42,43], a transcription factor that is closely linked to Akt, implying a potential association in the context of DHM. While our study primarily focuses on examining the role of the Sp1 transcription factor in the upregulation of involucrin mediated by Akt, it is evident that the promoter region of involucrin is affected by various elements. These elements include not only Sp1 but also molecules such as activator protein 1 and CCAAT/enhancer binding protein [44]. Therefore, the observed increase in involucrin activity, attributed to Akt elevation, may not be solely attributed to the involvement of Sp1. This is particularly evident considering that the administration of 50 μM of DHM can induce luciferase activity in the involucrin promoter without affecting Sp1 mRNA expression, suggesting the presence of additional mechanisms. It will be worthwhile to explore the downstream elements of Akt that are crucial in mediating the DHM-induced increase in involucrin levels and their individual contributions to the upregulation of involucrin.

It is critical to note that the skin barrier is composed of multiple proteins that function in tandem to regulate its function. For instance, loricrin, the predominant epidermal skin barrier protein (accounting for approximately 70% of the total), does not single-handedly determine barrier function, as evidenced by the fact that loricrin knockout mice maintain normal barrier function, implying that alterations in a single component may not trigger significant changes in the skin barrier [45]. In our study, we discovered that DHM enhanced the expression of involucrin in keratinocytes. However, it remains unclear whether the improvement in skin barrier function observed in the 3D human skin equivalent model was exclusively mediated by the upregulation of involucrin. Interestingly, the involucrin locus is situated within the epidermal differentiation complex on human chromosome 1q21, which comprises a cluster of more than 15 genes that are highly co-expressed during terminal differentiation, including filaggrin and loricrin [46,47]. We found that while DHM increased filaggrin expression, the Akt pathway was not involved in this increase, and DHM did not promote the effect of loricrin in keratinocytes. These findings suggest that the modulating effects of DHM on skin barrier genes are intricate, and not simply an all-or-none phenomenon. Therefore, a more comprehensive investigation is required to fully understand the overall effect of DHM on the skin barrier.

In this study, the effects of DHM were evaluated in both an in vitro setting and a 3D human skin model. While our findings provide valuable insights into the potential effects of DHM on skin barrier proteins and morphological changes, we acknowledge certain limitations inherent to our experimental approach. Although involucrin is widely recognized as a marker for skin barrier integrity and the correlation between involucrin levels and functional aspects of skin health has been established, our study did not extend to functional experiments that directly assess skin barrier properties such as moisture retention or transepidermal water loss (TEWL), which is necessary to provide direct experimental evidence for a more comprehensive understanding. Taking into account these limitations, future research should prioritize the incorporation of in vivo studies that allow for the direct measurement of functional parameters influenced by DHM.

## 4. Materials and Methods

### 4.1. Cell Culture

HaCaT cells were cultured in calcium-free DMEM/F-12 medium (Welgene, Seoul, Republic of Korea) supplemented with 10% fetal bovine serum (FBS; Gibco, Grand Island, NY, USA). The calcium in this medium was chelated using Chelex beads (Bio-Rad, Hercules, CA, USA). After chelation, the Chelex was removed using a 0.22 µm filter (Sartorius, Goettingen, Germany). The medium was further supplemented with antibiotics (100 µg/mL penicillin, 100 µg/mL streptomycin; Welgene) and CaCl_2_ was added to achieve a final concentration of 0.03 mM. NHEK cells were obtained from PromoCell (Heidelberg, Germany) and cultured in Keratinocyte Growth Medium 2 supplemented with a supplement mix (PromoCell). The cultures were maintained at 37 °C in a 5% CO_2_ humidified atmosphere. To induce differentiation, the basal medium in confluent HaCaT or NHEK cells was replaced with a high-calcium medium containing 1.8 mM CaCl_2_. The cells were subsequently incubated at 37 °C and 5% CO_2_ for the indicated time periods. If required, DHM (Sigma-Aldrich, St. Louis, MO, USA), LY294002 (MCE, Shanghai, China), or PP2 (MCE), was further administered at the indicated concentrations during the process of keratinocyte differentiation in the differentiating media.

### 4.2. Cell Viability Assay

Cell viability was assessed using a CCK-8 assay (Dojindo, Tokyo, Japan). HaCaT cells were cultured in 96-well plates for 24 h. The cells were then exposed to either DHM (25, 50, 100, 200, and 400 μM) or the vehicle control (dimethyl sulfoxide; DMSO; Sigma-Aldrich). After the treatment for 24, 48, and 72 h, the CCK-8 solution was added to each well, and the plates were incubated for 2 h. The absorbance values were measured at 450 nm wavelength using a microplate reader (Infinite 200 PRO NanoQuant; Tecan, Männedorf. Switzerland), and the corresponding optical density ratio was expressed as the cell viability.

### 4.3. Transfection and Luciferase Assay

HaCaT cells were seeded in 24-well culture plates at 80% confluence. Before transfection, the culture medium was removed, and 500 μL of antibiotic-free DMEM was added. Cells were co-transfected with the firefly luciferase plasmid encoding the promoter region of involucrin (~3.7 kb) [48,49] or Sp1 (SRE; Promega, Madison, WI, USA) and the plasmid containing a Renilla luciferase gene (Promega), using Lipofectamine 3000 transfection reagent (ThermoFisher, Waltham, MA, USA) according to the manufacturer’s protocol. Briefly, 350 ng of DNA was diluted in 25 μL of Opti-MEM (Gibco, Gaithersburg, MD, USA), and 0.75 μL of transfection reagent was combined with another 25 μL of Opti-MEM. The two dilutions mentioned above were mixed and incubated for 10 min at 25 °C, after which the complex was added to each well. Twenty-four hours post-transfection, DHM was added to the cell cultures for the indicated times. The cells were then washed with ice-cold PBS and harvested in 500 μL of passive lysis buffer (Promega). We used 10 μL of cell lysate for luminescence measurements using a GLOMAX luminometer (Promega) as follows: The firefly luciferase reagent (LARII; 50 μL; Promega) and the Renilla luciferase reagent (Stop&Glo; 50 μL; Promega) were sequentially added to the sample aliquot, and the luminescence of each reagent was measured. The activity of firefly luciferase was normalized to that of Renilla luciferase, which served as an internal transfection control.

### 4.4. RT-qPCR

The cells were isolated using TRIzol reagent (Invitrogen, Carlsbad, CA, USA), followed by chloroform extraction and isopropanol precipitation. The RNA was eluted in nuclease-free water and quantified using a NanoDrop spectrophotometer (Tecan). Reverse transcription was performed according to the manufacturer’s instructions using a PrimeScript RT Reagent Kit with gDNA Eraser (Takara, Tokyo, Japan). qPCR was performed in a final volume of 20 μL, containing 10 μL of TB Green Premix Ex Taq II (Takara), 0.3 μM of each primer, and 10 ng of complementary DNA. Initial denaturation at 95 °C for 30 s was followed by 35 cycles of denaturation at 95 °C for 5 s, and annealing and extension at 60 °C for 30 s. The 2^−ΔCt^ method was used to analyze the data, and the expression of each gene was normalized to that of glyceraldehyde 3-phosphate dehydrogenase (GAPDH). The primer sequences are shown in Table S1.

### 4.5. Western Blot

The cells were harvested and lysed using a protein extraction solution (PRO-PREP; iNtRON, Seoul, Republic of Korea). Following lysis, the cells were centrifuged at 13,000× *g* for 15 min at 4 °C. From the lysates, 25 μg of protein was isolated and separated on 10% polyacrylamide gels. The proteins were then transferred to nitrocellulose membranes (Whatman, Maidstone, UK) by electrophoresis. The membranes were blocked with 5% bovine serum albumin (BSA) in Tris-buffered saline containing 0.1% Tween-20 (TBST) to prevent non-specific binding. The membranes were then incubated with primary antibodies (Cell Signaling, Danvers, MA, USA) for 1 h at room temperature. Subsequently, the blots were rinsed three times with TBST solution and incubated for another hour at 20 °C with horseradish peroxidase-conjugated secondary antibodies (Sigma-Aldrich). After three additional washes with TBST, the blots were developed using an electrochemiluminescence detection reagent (Biomax, Seoul, Republic of Korea) and scanned using a light-capture system (ATTO, Tokyo, Japan). Specific band intensities were quantified and normalized using GAPDH as an internal control.

### 4.6. siRNA

HaCaT cells were seeded at a density of 1 × 10^5^ cells per well in 24-well plates and incubated for 24 h. Subsequently, the cells were transfected with either non-targeting siRNA or siRNA targeting the Akt or Fyn gene (Bionics, Seoul, Republic of Korea). The siRNAs were administered at a concentration of 50 nM using Opti-MEM as the transfection medium. The transfection protocol, using RNAiMAX (Invitrogen) as the transfection reagent, was conducted according to the manufacturer’s guidelines. After transfection, cells were incubated for an additional 48 h. The siRNA sequences used in this study were as follows: for Akt, the sense sequence was 5′-AGCGACGUGGCUAUUGUGATT-3′ and the anti-sense sequence was 5′-UCACAAUAGCCACGUCGCUTT-3′; for Fyn, the sense sequence was 5′-GGUGGAUACUACAUUACCATT-3′ and the anti-sense sequence was 5′-UGGUAAUGUAGUAUCCACCTT-3′. To assess the efficiency of siRNA-mediated knockdown, RT-PCR analysis was performed.

### 4.7. RNA Sequencing and Bioinformatics

The extraction of RNA was carried out as described above. The QuantSeq 3′ mRNA-Seq Library Prep Kit (Lexogen, Vienna, Austria) was used to construct the library. Briefly, total RNA was hybridized to an oligo-dT primer containing an Illumina-compatible sequence at its 5′-end to facilitate reverse transcription. Following RNA template degradation, second-strand synthesis was initiated using a random primer with an Illumina-compatible linker sequence at its 5′ end. The double-stranded library of reaction components was then purified using magnetic beads. The library was amplified to incorporate cluster-generating adapter sequences and subsequently purified using PCR. High-throughput single-end 75 sequencing was conducted using a NextSeq 500 (Illumina, San Diego, CA, USA). The QuantSeq 3′ mRNA-Seq reads were aligned using Bowtie2. The Bowtie2 indices were used to align the genome and transcriptome using either the genome assembly sequence or representative transcript sequences. The alignment file was used to assemble transcripts, estimate abundance, and detect differences in gene expression. We identified a list of genes whose expression was increased by DHM treatment and inferred upstream kinases by submitting the list of genes in the KEA3 web server application [50]. The top ten ranked kinases using the MeanRank score are reported. The lower value indicates a higher degree of significant enrichment for kinase substrate overlap between the query set and a gene set in the library.

### 4.8. Reconstructed Human Skin Model

The Neoderm-ED (Epidermis + Dermis) human skin equivalent (HSE) model was purchased from Tego Science (Seoul, Republic of Korea) and was placed in an incubator at 37 °C in a 5% CO_2_ atmosphere. The HSE was treated with DHM, PP2, or LY290042 and then incubated for 4 days. The skin equivalent was subsequently isolated from the culture plate, fixed in 10% formalin, and embedded in paraffin. Representative sections (5 μm thickness) were stained with hematoxylin and eosin. The samples were examined using a Nikon Eclipse TS2 microscope equipped with a DMX1200 camera (Nikon, Tokyo, Japan).

### 4.9. Statistical Analysis

Data are presented as mean ± standard error of the mean (SEM). The differences between the two groups were analyzed using SPSS (IBM, Version 26, Armonk, NY, USA) with a two-tailed Student’s *t*-test. The significance level was set at *p* < 0.05.

## 5. Conclusions

In summary, we demonstrated the increasing effect of the natural compound DHM on involucrin expression in keratinocytes and further confirmed this phenomenon in 3D human skin models. The effect of DHM is mediated by the Akt pathway, with Fyn playing a significant role as an upstream factor (Figure S4). Therefore, regulating involucrin expression using DHM could be a potential therapeutic strategy to improve barrier function. Moreover, this study suggests the possibility of barrier improvement by manipulating the Akt pathway and provides a scientific basis for the importance of Akt modulation in skin barrier control.

## Data Availability

The data that support the findings of this study are available from the corresponding author upon reasonable request.

## Supplementary Materials

The following supporting information can be downloaded at: https://www.mdpi.com/article/10.3390/ijms25042246/s1.
