# Supplementary material for: Dihydromyrcenol Modulates Involucrin Expression through the Akt Signaling Pathway
Source: Int J Mol Sci. 2024 Feb 13;25(4):2246. doi: 10.3390/ijms25042246 (PMC10889318; doi:10.3390/ijms25042246)
Supplement: Supplementary file 1 [file ijms-25-02246-s001.zip › Supplementary figure.pdf]

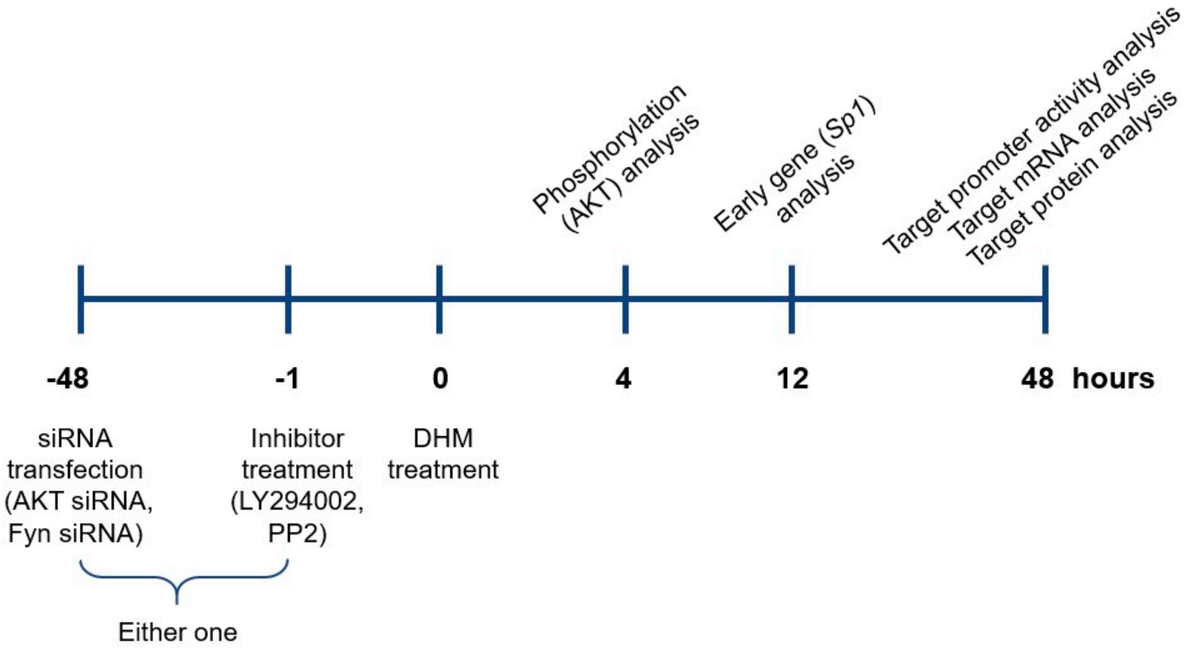

Supplementary Figure S1. Experimental design schematic. HaCaT cells underwent the following treatments: Transfection with siRNA targeting Akt or Fyn for a duration of 48 hours, or pretreatment with inhibitor (LY294002 or PP2) for a duration of 1 hour. Following treatment with dihydromyrcenol (DHM), the phosphorylation of Akt was measured at 1-4 hour time points. The levels of Sp1 mRNA were assessed after 12 hours, whereas promoter activity, involucrin mRNA, and protein levels were evaluated after 48 hours of DHM treatment in HaCaT cells.

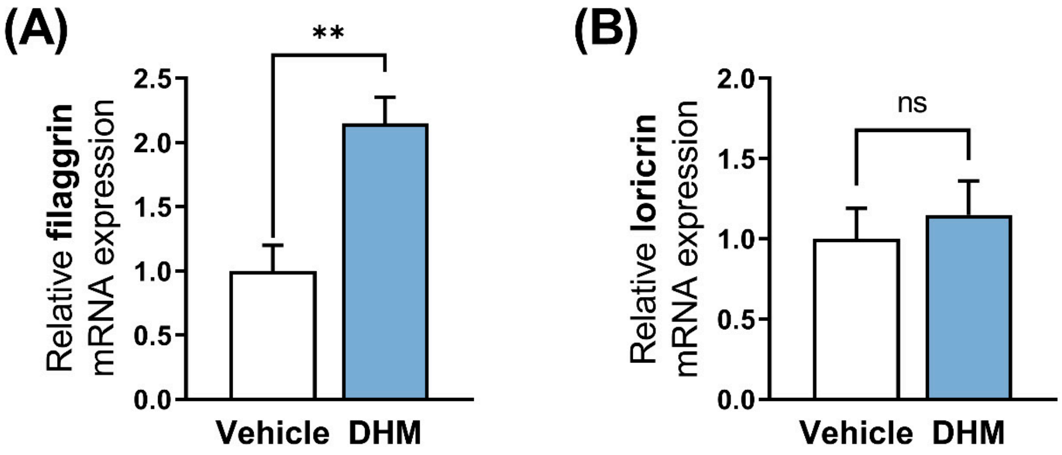

Supplementary Figure S2. DHM increased filaggrin expression but did not significantly alter the mRNA levels of loricrin in HaCaT keratinocytes. (A, B) During the differentiation process, HaCaT cells were treated with DHM (200 μM) for 48 hours. The expression of filaggrin and loricrin was assessed using RT-qPCR. Data are presented as the mean ± SEM, derived from three independent experiments. ** $p<0.01$; ns, not significant.

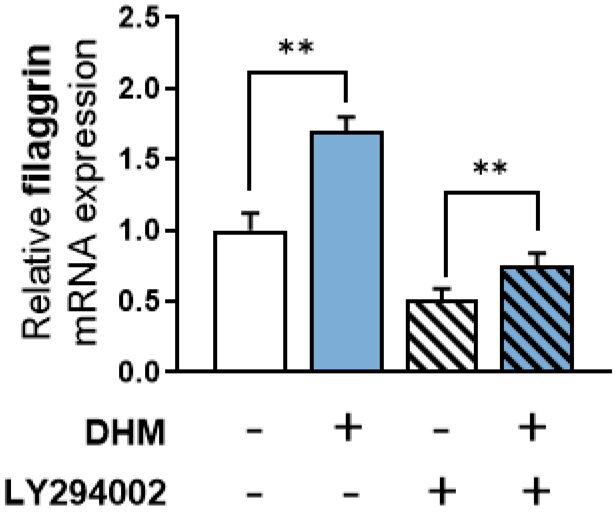

Supplementary Figure S3. The enhancement of filaggrin expression by DHM was not counteracted by LY294002. HaCaT cells were pre-treated with LY294002 (50 μM) for one hour before incubation with DHM (200 μM) for 48 hours. Filaggrin mRNA expression was subsequently measured using RT-qPCR. Data are presented as mean ± SEM, derived from three independent experiments. ** $p < 0.01$.

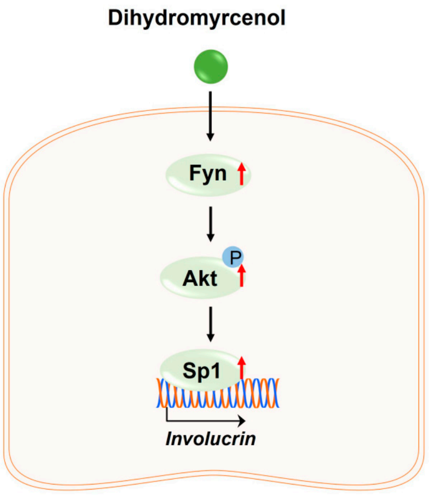

Supplementary Figure S4. Schematic illustration of the molecular mechanism of dihydromyrcenol-mediated involucrin upregulation in human keratinocytes.
