# Supplementary material for: Dihydromyrcenol Modulates Involucrin Expression through the Akt Signaling Pathway
Source: Int J Mol Sci. 2024 Feb 13;25(4):2246. doi: 10.3390/ijms25042246 (PMC10889318; doi:10.3390/ijms25042246)
Supplement: Supplementary file 1 [file ijms-25-02246-s001.zip › Supplementary Table S1.pdf]

Supplementary Table S1. Primer sequences for RT-qPCR

Gene description	Sequence (5′→3′)
Filaggrin	F: AAGCACAGAAAGCACAGTCATC<br>R: TTCTCTTGGGCTCTTGGATCTTC
Involucrin	F: TATTTCGGGTCCGCTAGGTG<br>R: ATTGGGGTCATTGGGGTTGG
Loricrin	F: CCTACCTGGAAGAAGCCATTGAG<br>R: ACAGAAACCAAAGAGGCTAAACAG
Sp1	F: GGTTCGCTTGCCTCGTCAG<br>R: GATCTTGGTCGCTCATGGTGG
Glyceraldehyde-3-phosphate dehydrogenase (*GAPDH*)	F: TCTGGAAAGCTGTGGCGTGA<br>R: TACTTGGCAGGTTTCTCCAGG
